# Posttraumatic stress among women after induced abortion: a Swedish multi-centre cohort study

**DOI:** 10.1186/1472-6874-13-52

**Published:** 2013-12-23

**Authors:** Inger Wallin Lundell, Susanne Georgsson Öhman, Örjan Frans, Lotti Helström, Ulf Högberg, Sigrid Nyberg, Inger Sundström Poromaa, Gunilla Sydsjö, Ingrid Östlund, Agneta Skoog Svanberg

**Affiliations:** 1 Department of Women’s and Children’s Health, Uppsala University, SE-751 85 Uppsala, Sweden; 2 Sophiahemmet University, Box 5605, SE-114 86, Stockholm, Sweden; 3 Department of Women’s and Children’s Health, Karolinska Institutet, SE-171 77 Stockholm, Sweden; 4 Department of Psychology, Uppsala University, Box 1225, SE-751 42 Uppsala, Sweden; 5 Department of Clinical Science and Education, Karolinska Institutet, SE-118 83 Stockholm, Sweden; 6 Department of Clinical Sciences, Obstetrics and Gynaecology, Umeå University, SE-901 87 Umeå, Sweden; 7 Division of Obstetrics and Gynaecology, Department of Clinical and Experimental Medicine, Faculty of Health Sciences, Linköping University, SE-581 85 Linköping, Sweden; 8 Department of Obstetrics and Gynaecology, Örebro University Hospital, SE-701 85 Örebro, Sweden

**Keywords:** Induced abortion, Posttraumatic stress disorder, Anxiety disorders, Mental health

## Abstract

**Background:**

Induced abortion is a common medical intervention. Whether psychological sequelae might follow induced abortion has long been a subject of concern among researchers and little is known about the relationship between posttraumatic stress disorder (PTSD) and induced abortion. Thus, the aim of the study was to assess the prevalence of PTSD and posttraumatic stress symptoms (PTSS) before and at three and six months after induced abortion, and to describe the characteristics of the women who developed PTSD or PTSS after the abortion.

**Methods:**

This multi-centre cohort study included six departments of Obstetrics and Gynaecology in Sweden. The study included 1457 women who requested an induced abortion, among whom 742 women responded at the three-month follow-up and 641 women at the six-month follow-up. The Screen Questionnaire-Posttraumatic Stress Disorder (SQ-PTSD) was used for research diagnoses of PTSD and PTSS, and anxiety and depressive symptoms were evaluated by the Hospital Anxiety and Depression Scale (HADS). Measurements were made at the first visit and at three and six months after the abortion. The 95% confidence intervals for the prevalence of lifetime or ongoing PTSD and PTSS were calculated using the normal approximation. The chi-square test and the Student’s t-test were used to compare data between groups.

**Results:**

The prevalence of ongoing PTSD and PTSS before the abortion was 4.3% and 23.5%, respectively, concomitant with high levels of anxiety and depression. At three months the corresponding rates were 2.0% and 4.6%, at six months 1.9% and 6.1%, respectively. Dropouts had higher rates of PTSD and PTSS. Fifty-one women developed PTSD or PTSS during the observation period. They were young, less well educated, needed counselling, and had high levels of anxiety and depressive symptoms. During the observation period 57 women had trauma experiences, among whom 11 developed PTSD or PTSS and reported a traumatic experience in relation to the abortion.

**Conclusion:**

Few women developed PTSD or PTSS after the abortion. The majority did so because of trauma experiences unrelated to the induced abortion. Concomitant symptoms of depression and anxiety call for clinical alertness and support.

## Background

Induced abortion is a common medical intervention in countries where legal abortion is generally available. In 2008, approximately 20% of all known pregnancies ended in abortion in the United States, England/Wales and Sweden. Sweden has the highest abortion rate in the Nordic countries, and in all of Europe only some countries in Eastern Europe report higher abortion rates than Sweden [1]. Nine out of ten abortions before the end of gestational week nine are medical abortions in Sweden [2].

Whether psychological sequelae might follow induced abortion has long been a subject of concern among researchers. Longitudinal prospective studies of the mental health of women who have had an induced abortion are important but scarce [3] and methodological problems are common. The recruitment of participants is generally difficult with response rates as low as 47%, and sample sizes have in most studies been inadequate [4-6]. In addition, response rates at follow-up reportedly vary between 50–65% [7,8], and dropouts appear more vulnerable to mental health issues than responders [8]. Thus, careful dropout analyses are necessary for the overall interpretation of the study results [7]. Existing data on the mental health of women who have had an induced abortion have yielded negative findings, that is, no association between the induced abortion and subsequent psychological sequelae [3,9,10]. Instead, the strongest predictor of a woman’s mental health after induced abortion is her mental health prior the abortion [7,11].

Posttraumatic stress disorder (PTSD) is relatively common in the general female population [12,13]. Genetic vulnerability, personality factors and ongoing mental illness may predispose one to the development of PTSD or posttraumatic stress symptoms (PTSS) [14-17]. In an earlier cross -sectional study among 1514 abortion-seeking women in Sweden the lifetime and the point prevalence of PTSD were 7% and 4%, respectively, and the prevalence of PTSS was 23% at the time of the abortion [18].

A research study employing a prospective design is the only way to determine possible changes in mental health, such as PTSD, in relation to induced abortion. Thus, the aim of this study was to assess the prevalence of PTSD and PTSS before induced abortion and then at three and six months after the abortion. An additional aim was to describe the characteristics of women who did develop PTSD or PTSS after the abortion.

## Methods

Between September 2009 and June 2010, a multi-centre study targeting women who requested an induced abortion was conducted at the outpatient clinics of the Departments of Gynaecology and Obstetrics of six public hospitals in Sweden. All women who requested an induced abortion before the end of gestational week 12 were approached for participation in the study and the only exclusion criterion for the study was an inability to read and to understand Swedish.

Women were informed about the study by research nurses or midwives at registration for the first abortion visit. Women who agreed to participate received written information together with a questionnaire (baseline assessment). They were asked to sign an informed consent and fill out the questionnaire. Upon completion, questionnaires were deposited in a locked mailbox. Briefly, 2602 women were invited to participate and 1514 women consented and filled out the baseline questionnaire (overall response rate 58.2%, response rate per clinic 45–77%). Among these 1514 women, 44 had not filled out the entire SQ-PTSD, leaving 1470 women available for evaluation of PTSD or PTSS research diagnoses. The 1470 women did all request an abortion but thirteen were excluded because they did not have an abortion or had a second trimester abortion.

Two follow-up questionnaires were sent by post to participating women, at three and six months after the abortion, with two reminders for each questionnaire. Women who completed all three questionnaires received two cinema tickets.

### Measurements

The following instruments were used at all three assessments: The Screen Questionnaire - Posttraumatic Stress Disorder (SQ-PTSD) [13] and the Hospital Anxiety and Depression scale (HADS) [19]. The baseline questionnaire also contained questions on sociodemographic variables such as age, marital status, education, ethnicity, and tobacco and alcohol use. Supplementary information was retrieved from the medical records including parity, numbers of previous abortions, abortion method, place of abortion (home or at the clinic), antidepressant use, and psychosocial support during the abortion process.

The SQ-PTSD is based on the Diagnostic and Statistical Manual of Mental Disorders, Fourth edition (DSM-IV) diagnostic criteria for PTSD, and assesses trauma experiences as well as trauma symptoms. The DSM-IV criteria are: A1) Confrontation with the stressor should involve actual or threatened death or serious injury, or a threat to the physical integrity of self or others; A2) the response to the confrontation should involve fear, helplessness or horror; B) persistent re-experiencing of the traumatic event in intrusive thoughts, nightmares or flashbacks; C) persistent avoidance of stimuli associated with the event and emotional numbing symptoms, described as an inability to experience any positive feelings such as love, contentment, satisfaction and happiness; D) hyper arousal symptoms such as difficulties in sleeping, concentrating and controlling anger; E) duration of the disturbance (symptoms of criteria B, C, and D) for more than one month; and F) the disturbance causes clinically significant distress or impairment in social and occupational, or other important areas of functioning [20].

Only women who met all of the DSM-IV criteria from A to F were classified as having a research diagnosis of PTSD. Ongoing PTSD was measured with an additional question asking “if the trauma symptoms are present right now”.

Different terms have been used to denote individuals who only partly meet the diagnostic criteria of PTSD: sub-threshold PTSD, partial PTSD, or posttraumatic stress symptoms (PTSS) [21]. In the present study, we used the term PTSS, which was defined as prevalence of A1 and A2 criteria together with one or more of the re-experiencing, avoidance or hyper arousal symptoms (B-C-D criteria).

The Hospital Anxiety and Depression scale (HADS) measures anxiety and depressive symptoms and contains 14 items divided into two scales: Seven items evaluating anxiety and seven evaluating depressive symptoms [19]. The instrument has been validated in several clinical populations with satisfactory results [22] and for Swedish circumstances [23]. Depressive and anxiety symptoms are defined by the HADS questionnaire as: none (score 0–6); depressive mood/mild or moderate anxiety (score 7–10); and risk for depression/possible anxiety disorder (score >10).

### Statistical analysis

Sociodemographic data were categorised as follows: Age 15–19, 20–24, 25–34 and 35 years and older; marital status as cohabiting or not; education as less than 12 years (high school not completed) or more than 12 years of education; occupation as full-time, part-time, student or other occupation. Alcohol use was categorised as use or no use, and country of birth as native born Swede or not, tobacco use as smoking and/or snuff use. Anxiety and depressive scores on the HADS were categorised as none (score 0–7) or present (score 8–21), to clarify whether anxiety and depression symptoms were present or not. Data from the medical records were categorised as having children (yes/no), previous abortion (yes/no), and counselling before and after abortion (yes/no). The 95% confidence intervals for the prevalence of lifetime or ongoing PTSD and PTSS were calculated using the normal approximation.

The responders were categorised into four groups, depending on time-course of PTSD or PTSS research diagnoses: 1) Women who had *no* PTSD or PTSS at baseline but met the criteria for PTSD or PTSS at least once at the three or six-month assessments were classified as having *developed PTSD or PTSS*. Women who had PTSS at baseline but met the criteria for PTSD at least once at the three- or six-month assessments were also included in this group. 2) Women who had PTSD or PTSS at baseline but no longer met the criteria for PTSD or PTSS, respectively, at the three or six-month assessments were classified as *recovered*. Women who had PTSD or PTSS at baseline, missing data at the three-month assessment but no longer met the criteria for PTSD or PTSS, respectively, at the six-month assessment were also assigned to this group. Of note, at the six-month assessment, these women did not fulfil criteria for either PTSD or PTSS. 3) Women who met criteria for PTSD or PTSS at all assessments were classified as *unchanged*. 4) Finally, women who never fulfilled criteria for PTSD or PTSS at any time-point were used as a *comparison group.* Women with missing data for PTSD/PTSS at baseline were excluded.

The sociodemographic data, reproductive history, and prevalence of PTSD and PTSS were used to explore differences in characteristics between responders and dropouts at the three and six-month assessments. The chi-square test and Student’s t-test were used and the last available information was included in the analyses, that is, the data on dropouts at the three-month assessment were derived from the baseline questionnaire, and data on the dropouts at the six-month assessment was derived from the three-month questionnaire. Similarly, the chi-square test was used to compare sociodemographic data and reproductive history between women who developed PTSD or PTSS, recovered from PTSD or PTSS, remained unchanged in their state of PTSD or PTSS, and the comparison group. A P-value at 0.05 was used as the threshold for statistical significance, and IBM SPSS Statistics for Windows, Version 20.0. (IBM Corp, Armonk, NY) was used for statistical analysis.

### Details of ethical approval

The study has been approved by the Independent Research Ethics Committee at Uppsala University, dnr 2009/012. Approval 25 of February 2009.

## Results

**Figure 1:**
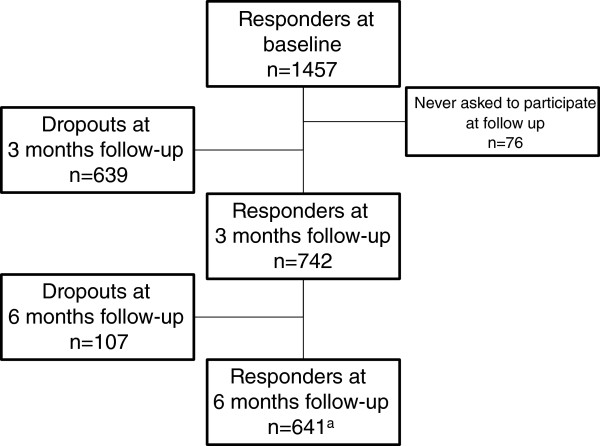
**Flowchart of the responders at baseline, and responders and dropouts at 3 & 6 months. **^a^including six responders who were non-responders at the three-month assessment.

Of 1457 women who were qualified responders at baseline, 76 were never reported to the study centre and consequently never received the follow-up questionnaire. Response rates were 742/1381 (54%) at the three-month follow-up and 641/1381 (46%) at the six-month assessment (Figure 1).

Dropouts at the three-month assessments were younger, more often born outside Sweden, had a lower level of education, reported tobacco use more often but less alcohol use, had more anxiety and depressive symptoms and were more often using antidepressant treatment. In addition, they had more often had a previous abortion and had less often received counselling before the abortion (Table 1), and they also had higher rates of lifetime PTSD, ongoing PTSD and PTSS at the baseline assessment than the responders (Table 2). Dropouts at the six-month assessment had lower levels of education and had more often had a previous induced abortion (Table 1), but did not differ from responders in rates of lifetime PTSD, ongoing PTSD or PTSS (Table 2).

**Table 1:** Characteristics of responders and dropouts (in italics) at the first visit at the clinic (baseline) and at follow-up after the abortion

**Variable**	**Baseline responders**				**Responders at 3 month**		
	**All**	**Responders 3 month follow-up**	***Dropouts 3 month follow-up***^***1***^		**Responders 6 month follow-up**^**2**^	***Dropouts 6 month follow-up***^***2***^	
	**n = 1457**	**n = 742**	***n = 639***		**n = 641**^**3**^	***n = 107***	
	**n (%)**	**n (%)**	***n (%)***	***P-*** **value**	**n (%)**	***n (%)***	***P*-value**
**Age**				0.001			0.8
15–19	135 (9.3)	57 (7.7)	*70 (11.0)*		49 (7.7)	*9 (8.4)*	
20–24	443 (30.4)	208 (28.1)	*217 (34.0)*		176 (27.5)	*34 (31.8)*	
25–34	564 (38.7)	293 (39.5)	*237 (37.1)*		254 (39.7)	*41 (38.3)*	
35–	314 (21.6)	183 (24.7)	*115 (18.0)*		161 (25.2)	*23 (21.5)*	
Mean ± SD	28.0 ± 7.2	28.7 ± 7.4	*27.1 ± 6.9*	<0.001	28.8 ± 7.5	*27.9 ± 7.1*	0.3
**Living alone**	379 (27.0)	182 (25.2)	*174 (28.6)*	0.2	152 (24.4)	*31 (29.8)*	0.3
**Education <12 years**	1009 (69.8)	465 (63.2)	*493 (77.9)*	<0.001	392 (61.6)	*78 (73.6)*	0.018
**Occupation**				0.06			0.1
Working full-time	572 (40.6)	297 (41.2)	*240 (39.2)*		250 (40.1)	*48 (46.2)*	
Working part-time	270 (19.2)	150 (20.8)	*104 (17.0)*		139 (22.3)	*12 (11.5)*	
Student	355 (25.2)	179 (24.8)	*160 (26.1)*		152 (24.4)	*28 (26.9)*	
Other occupation	211 (15.0)	95 (13.2)	*109 (17.8)*		82 (13.2)	*16 (15.4)*	
**Anxiety symptoms**	622 (43.5)	283 (38.6)	*302 (48.6)*	<0.001	190 (30.3)	*30 (28.3)*	0.7
**Depression symptoms**	449 (31.4)	210 (28.6)	*210 (33.8)*	0.043	85 (13.6)	*14 (13.2)*	1.0
**Antidepressant use**	98 (7.2)	40 (5.6)	*53 (9.2)*	0.012	36 (5.8)	*5 (4.9)*	0.8
**Alcohol use**	1154 (79.7)	607 (82.1)	*486 (76.8)*	0.014	519 (81.2)	*91 (85.8)*	0.3
**Tobacco use**	573 (39.5)	249 (33.7)	*297 (46.8)*	<0.001	206 (32.3)	*45 (42.5)*	0.040
**Foreign born**	120 (8.3)	45 (6.1)	*60 (9.5)*	0.019	41 (6.4)	*4 (3.8)*	0.3
**Having no children**	743 (55.9)	384 (54.5)	*314 (56.2)*	0.6	333 (54.8)	*53 (52.5)*	0.7
**Previous abortion**	505 (39.3)	232 (34.0)	*242 (44.9)*	<0.001	186 (31.7)	*47 (47.0)*	0.003
**Abortion method**				0.7			0.2
Medical	1103 (81.5)	594 (83.0)	*467 (81.9)*		520 (84.0)	*79 (77.5)*	
Surgical	251 (18.5)	122 (17.0)	*103 (18.1)*		99 (16.0)	*23 (22.5)*	
**Place for abortion**				0.8			1.0
Home	716 (53.0)	396 (55.4)	*309 (54.5)*		344 (55.6)	*56 (55.4)*	
Clinic	643 (47.0)	319 (44.6)	*258 (45.5)*		275 (44.4)	*45 (44.6)*	
**Counselling before abortion**	515 (40.4)	308 (45.7)	*204 (38.3)*	0.01	272 (46.5)	*38 (40.4)*	0.3
**Counselling after abortion**	36 (2.8)	20 (3.0)	*13 (2.5)*	0.6	19 (3.3)	*1 (1.1)*	0.2

Frequencies are given in relation to available information or responses. Missing information was prevalent in 0.07 (age) –13% (Counselling after the abortion) of variables.

^1^Analysed with chi-square test based on data from the baseline questionnaire.

^2^Analysed with chi-square test based on data from the three-month assessment.

^3^Including six responders who were non-responders at 3 month follow-up.

**Table 2:** Prevalence of lifetime PTSD, PTSS and ongoing PTSD at the first visit to the clinic (baseline), and at follow-up

**Category**	**Baseline responders**				**Responders at 3 month**				**Responders at 6 month**
	**All**	**Responders at 3 month follow-up**^**1**^	***Dropouts at 3 month follow-up***^***1***^		**All**	**Responders at 6 month follow-up**^**2**^	***Dropouts at 6 month follow-up***^***2***^		**All**
	**n = 1402/1457**	**n = 714/742**	***n = 616/639***		**n = 736/742**	**n = 629/641**	***n = 107/107***		**n = 641**^**3**^**/64**
	**n (%)**	**n (%)**	***n (%)***	***P-*** **value**	**n (%)**	**n (%)**	***n (%)***	***P*-value**	**n (%)**
	**((95% CI))**				**((95% CI))**				**((95% CI))**
Lifetime PTSD	101 (7.2)	36 (5.0)	*60 (9.7)*	<0.001	21 (2.9)	17 (2.7)	*4 (3.7)*	0.7	15 (2.3)
	((5.9–8.6))				((1.7-4.1))				((1.1–3.5))
PTSS	329 (23.5)	148 (20.7)	*161 (26.1)*	<0.001	34 (4.6)	28 (4.5)	*6 (5.6)*	0.7	39 (6.1)
	((21.2–25.7))				((3.1-6.1))				((4.2–8.0))
Ongoing PTSD^4^	60 (4.3)	21 (2.9)	*38 (6.2)*	0.004	15 (2.0)	12 (1.9)	*3 (2.8)*	0.5	12 (1.9)
	((3.2–5.4))				((1.0-3.0))				((0.8–3.0))

CI, confidence interval. Dropout analysis at baseline and at 3 months derived by comparing responders with dropouts at baseline and at 6 months respectively.

^1^Analysed with chi-square test based on data from the baseline questionnaire.

^2^Analysed with chi-square test based on data from three-month assessment.

^3^Including six responders who were non-responders at 3 month follow-up.

^4^Missing variables for ongoing PTSD was < 1%.

The prevalence of lifetime PTSD at baseline was 7.2%, at three months 2.9% and at six months 2.3%. The prevalence of PTSS at baseline was 23.5%, at three months 4.6% and at six months 6.1%. The prevalence of ongoing PTSD at baseline was 4.3%, at three months 2% and at six months 1.9% (Table 2).

**Figure 2:**
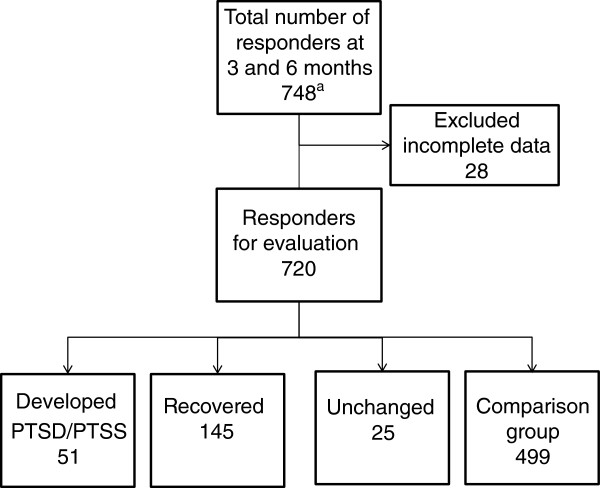
**Flowchart of the time-course of PTSD or PTSS research diagnoses. **^a^including six responders who were non-responders at the three-month assessment.

Table 3 displays the demographic characteristics of women who developed (n = 51), recovered (n = 145) or remained unchanged (n = 25) in their PTSD or PTSS status during the observation period in comparison with the comparison group (n = 499) (see also Figure 2). Women who developed PTSD or PTSS were to a greater extent younger, more often students, less often working full-time, had a lower level of education, did not have children, and had more often received counselling before the abortion. However, the abortion method or the place of abortion was not associated with development of PTSD or PTSS (Table 3). The comparison group had lower levels of anxiety and depressive symptoms throughout the study than those who developed, recovered or remained unchanged in their state of PTSD or PTSS. Women who remained unchanged in their PTSD or PTSS during the observation period had the highest rates of depression and anxiety at all three assessment points (Table 4).

**Table 3:** Characteristics of women who developed PTSD or PTSS after abortion compared with the comparison group

**Variable**	**Comparison group n = 499**	**Developed PTSD/PTSS n = 51**		***Recovered n = 145***		***Unchanged n = 25***	
	**n (%)**	**n (%)**	***P*-value**	***n (%)***	***P-*** **value**	***n (%)***	***P-*** **value**
**Age**			0.001		*0.7*		*0.1*
15–19	33 (6.6)	7 (13.7)		*13 (9.0)*		*4 (16.0)*	
20–24	129 (25.9)	24 (47.1)		*41 (28.3)*		*4 (16.0)*	
25–34	204 (40.9)	15 (29.4)		*57 (39.3)*		*14 (56.0)*	
34–	133 (26.7)	5 (9.8)		*34 (23.4)*		*3 (12.0)*	
**Living alone**	119 (24.3)	17 (34.7)	0.2	*33 (23.6)*	*0.9*	*6 (24.0)*	*1.0*
**Education <12 years**	297 (59.8)	37 (74.0)	0.049	*101 (69.7)*	*0.031*	*19 (76.0)*	*0.1*
**Occupation**			0.01		*0.2*		*0.2*
Working full-time	222 (45.5)	10 (20.8)		*51 (35.9)*		*6 (24.0)*	
Working part-time	96 (19.7)	13 (27.1)		*32 (22.5)*		*5 (20.0)*	
Student	106 (21.7)	17 (35.4)		*41 (28.9)*		*8 (32.0)*	
Other occupation	64 (13.1)	8 (16.7)		*18 (12.7)*		*6 (24.0)*	
**Alcohol use**	416 (83.4)	37 (72.5)	0.1	*117 (80.7)*	*0.5*	*18 (72.0)*	*0.2*
**Tobacco use**	152 (30.5)	18 (35.3)	0.5	*54 (37.2)*	*0.2*	*16 (64.0)*	*<0.001*
**Foreign born**	27 (5.4)	3 (5.9)	0.9	*13 (9.0)*	*0.2*	*1 (4.0)*	*0.8*
**Having no children**	257 (53.4)	32 (72.7)	0.014	*75 (55.1)*	*0.7*	*11 (45.8)*	*0.5*
**Previous abortion**	147 (31.6)	17 (38.6)	0.4	*49 (37.7)*	*0.2*	*12 (48.0)*	*0.1*
**Abortion method**			0.7		*0.9*		*1.0*
Medical	404 (83.3)	38 (80.9)		*116 (82.9)*		*21 (84.0)*	
Surgical	81 (16.7)	9 (19.1)		*24 (17.1)*		*4 (16.0)*	
**Place for abortion**			0.4		*0.3*		*0.6*
Home	279 (57.6)	23 (51.1)		*73 (51.8)*		*13 (52.0)*	
Clinic	205 (42.4)	22 (48.9)		*68 (48.2)*		*12 (48.0)*	
**Counselling before abortion**	187 (41.6)	27 (60.0)	0.017	*75 (55.1)*	*0.005*	*11 (45.8)*	*0.7*
**Counselling after abortion**	10 (2.2)	3 (6.7)	0.1	*4 (3.0)*	*0.7*	*1 (4.2)*	*0.6*

The characteristics of women who recovered from PTSD or PTSS or were unchanged in their state of PTSD or PTSS are displayed as complementary information, in italics.

Frequencies are given in relation to available information or responses. Missing information was prevalent in 0% (age) – 13.7% (previous abortion) of variables.

**Table 4:** Characteristics of women who developed PTSD or PTSS after the induced abortion

**Variable**	**Comparison group n = 499**	**Developed PTSD/PTSS n = 51**		***Recovered n = 145***		***Unchanged n = 25***	
	**n (%)**	**n (%)**	***P*-value**	**n (%)**	***P-*** **value**	**n (%)**	***P*-value**
**Anxiety symptoms at baseline**	139 (28.0)	32 (62.7)	<0.001	*86 (60.6)*	*<0.001*	*19 (76.0)*	*<0.001*
**Anxiety symptoms at 3 months**	99 (20.2)	26 (52.0)	<0.001	*67 (47.2)*	*<0.001*	*21 (87.5)*	*<0.001*
**Anxiety symptoms at 6 months**	83 (19.6)	27 (56.2)	<0.001	*62 (49.2)*	*<0.001*	*16 (88.9)*	*<0.001*
**Depressive symptoms at baseline**	113 (22.8)	18 (35.3)	0.046	*54 (38.0)*	*<0.001*	*20 (80.0)*	*<0.001*
**Depressive symptoms at 3 months**	34 (7.0)	16 (32.0)	<0.001	*34 (23.9)*	*<0.001*	*13 (54.2)*	*<0.001*
**Depressive symptoms at 6 months**	28 (6.6)	12 (25.0)	<0.001	*31 (24.6)*	*<0.001*	*9 (50.0)*	*<0.001*
**Antidepressant use**	16 (3.3)	4 (8.7)	0.1	*17 (12.1)*	*<0.001*	*3 (12.0)*	*0.025*

The characteristics of women who recovered from PTSD or PTSS or were unchanged in their state of PTSD or PTSS are displayed as complementary information, in italics. Frequencies are reported in relation to available responses for specific items and at specific time-points.

Trauma experiences during the period between the induced abortion and the three months assessment were reported by 57/720 women, or every 12^th^ woman. The most commonly reported traumas were physical and psychological threats from a partner or from other persons, followed by traumatic events in the family and accidents. Among the 57 women, 14 reported trauma experiences that were related to the abortion, without giving any examples of what kind of trauma they had experienced. Eleven of these developed PTSD or PTSS. Of the 14 women who reported trauma experiences related to the abortion, nine had had a medical and three had had a surgical abortion. Six women had their abortion performed at the clinic and six at home. Two had missing data of abortion method and place of abortion.

## Discussion

The major finding of the present study was that only a small fraction of women who have an induced abortion developed PTSD or PTSS. In addition, the majority of women who in fact developed PTSD or PTSS within a six- month period of the induced abortion did so because of trauma experiences unrelated to the induced abortion.

The lifetime prevalence of PTSD was 7.2% at the baseline assessment, which is somewhat lower than the 10.4% reported from the United States by Kessler and co-workers [12]. The result, however, is in accordance with the earlier reported population figure of 7.4% in Swedish women [13], indicating that women who request induced abortion do not suffer different rates of PTSD to the general population. There were two major reasons why lifetime prevalence of PTSD decreased over time, the most obvious reason was that a greater proportion of women with lifetime PTSD at baseline dropped out from the study. It should also be noted that lifetime PTSD was assessed by a questionnaire, not a structured psychiatric interview, and women may be inconsistent in their responses over time. The rates of ongoing PTSD at three and six-months after the induced abortion were 2.0% and 1.9%, respectively. This is in the lower end of what has been reported after childbirth, where PTSD prevalence rates of 1.3–6.9% have been found [24-26]. Although the rates reported in this study could be underestimated, still only a minority of those who reported a traumatic experience did so in relation to the abortion. The small number of women who developed PTSD or PTSS were more likely to be younger, less well educated, have high levels of anxiety and depression and in need of more counselling than the comparison group. Only 14 women, among whom 11 developed PTSD or PTSS, had trauma experiences related to the abortion, which suggests that women in general do not develop PTSD or PTSS following abortion.

Of 720 women, 51 developed PTSD or PTSS after the abortion, and they had a higher level of depression and anxiety, and needed counselling more often before the abortion than the comparison group. Ambivalence about the decision, strong maternal feelings, poor social support, moral and religious objections to abortion, coerced abortion, intimate partner violence and young age are all risk factors associated with adverse reactions after abortion [27,28]. In the present study, we have not explored the underlying causes for the women’s decision for the termination of the pregnancy because it was not a study aim. Also, in Sweden it is common practice not to ask women why they desire an induced abortion. However, mixed feelings are common as a natural reaction among women applying for abortion [28]. Feelings of guilt, sadness and regret only occur in a relatively small group of women and these feelings commonly arise in those who are ambivalent about the termination [10]. In addition, a previous study investigated the association of past elective or spontaneous abortions and mental health status during the subsequent pregnancy, suggesting that it is not the procedure of the abortion itself that increases the risk of PTSD but rather the women’s appraisal of the abortion.

In a Swedish longitudinal abortion study, 12 of 58 women reported in the four month follow- up that they had been in a crisis post abortion, while at the one-year follow- up, only two women still expressed feelings in relations to the abortion in terms of a crisis. Those in the study who reported only painful feelings at the time of the abortion decreased from 30% to 3% at one-year follow-up, while those who only reported positive feelings increased from 16% to 47% [28]. These results might be interpreted as resilience for self-healing and it is possible that women in the present study who reported mental disturbance may at one-year follow-up be healthier and report less disturbance.

In the present study, one-third of all women met criteria for PTSD or PTSS at least once during the observation period. Irrespective of whether their symptoms developed, recovered or were unchanged, these women displayed distinctly higher rates of anxiety and depressive symptoms than the comparison group did. This finding highlights how important it is that health care providers are responsive to women’s needs for support. Finally, all women who have PTSD at the time of the abortion as well as those who develop PTSD following the abortion need support. If these women can be identified in advance at the abortion clinics, they could be offered extra support and counselling before the abortion and this could be followed up by extra visits or telephone support after the abortion.

The main limitations for the interpretation of this study are the high rate of dropouts and their sociodemographic characteristics, which ultimately may suggest that they have an increased susceptibility to develop PTSD. The dropouts were younger than the responders, they had a lower level of education, displayed more anxiety and depression symptoms, were more often using antidepressant treatment, and were to a greater extent tobacco users. As expected, the social gradients of the dropouts are in line with earlier findings [8]. Indeed, the poorer mental health among the dropouts, including a higher baseline prevalence of PTSD, is a likely explanation for their decision not to participate further in the study. As the sociodemographic characteristics of the dropouts all are of importance for PTSD, it thus possible that the rates of PTSD and PTSS after induced abortion may be underestimated in this study. However, the rate of lifetime PTSD (9.4%) among dropouts was not higher than the population figure for American women (10.4%) [12]. Importantly, our findings are also in line with a number of studies suggesting that induced abortion is not associated with mental health problems. Although some researchers support the view that induced abortion is associated with an increased risk for mental health problems [29,30], the research supporting this view has been criticised for methodological errors, particularly in a recent meta-analysis [31]. Most studies report that women cope well with an induced abortion [28] and that psychological sequelae are rare [3,9,10]. In fact, a recent review further establishes that the most consistent predictor for mental disorders after induced abortion is the woman’s mental health prior to the abortion [32].

The study design also had several strengths such as the size of the study population and the multi-centre nationwide design, which allowed us to approach all women who requested an induced abortion at out-patient clinics representative of large and middle-sized cities all across the country. The comparison group of the present study had also undergone induced abortion, which is in contrast with many other studies in the field [3]. Another strength of the study is the use of a standardised and validated instrument for assessment of PTSD which takes the trauma experience as well as the trauma symptoms into account. The latter is important as our study suggested that many trauma experiences after the induced abortion were unrelated to the abortion care *per se*.

## Conclusion

Only a small fraction of women developed PTSD or PTSS at follow-up. The vast majority of those did so because of trauma experiences unrelated to the induced abortion. Concomitant symptoms of depression and anxiety call for clinical alertness and support.

## Abbreviations

CI: Confidence interval; DSM-IV: The Diagnostic and Statistical Manual of Mental Disorders, Fourth edition; HADS: The Hospital Anxiety and Depression Scale; PTSD: Posttraumatic stress disorder; PTSS: Posttraumatic stress symptoms; SQ-PTSD: Screen questionnaire-posttraumatic stress disorder.

## Competing interest

The authors declare that they have no competing interests.

## Authors’ contribution

IWL, data collection, analysis of data, preparation of the article, responsibility for the final preparation of the article. SGÖ, analysis of data, preparation of the final article. ÖF, research idea, design, preparation of the article. LH, research idea, design, data collection, preparation of the article. UH, research idea, design, data collection, analysis of data, preparation of the article. SN, collection of data, preparation of the article. ISP, research idea, design, analysis of data, final preparation of the article. GS, research idea, data collection, preparation of the article. IÖ, research idea, design, preparation of the article. ASS, research idea, design, data collection, analysis of data, final preparation of the article. All the authors have approved the final submitted version of the manuscript.

## Pre-publication history

The pre-publication history for this paper can be accessed here:

http://www.biomedcentral.com/1472-6874/13/52/prepub
